# The Association of Hemoglobin A1c Levels and Depression Among Adults With Diabetes in the United States

**DOI:** 10.7759/cureus.22688

**Published:** 2022-02-28

**Authors:** Joshua Langberg, Anna Mueller, Pura Rodriguez de la Vega, Grettel Castro, Marcia Varella

**Affiliations:** 1 Medicine, Florida International University, Herbert Wertheim College of Medicine, Miami, USA; 2 Medical and Population Health Sciences Research, Florida International University, Herbert Wertheim College of Medicine, Miami, USA

**Keywords:** hyperglycemia, hypoglycemia, depression screening, nhanes, hba1c, phq-9, depression, type 2 diabetes

## Abstract

Aim: Diabetes mellitus is linked to a decreased health-related quality of life, including poor mental health. Glycated hemoglobin/hemoglobin A1c (HbA1c) is an important marker in the diagnosis and management of diabetes mellitus. The main objective of this study was to assess the association between HbA1c levels (adequate control of serum glucose levels) and depression status among people with diabetes mellitus in the United States.

Methods: We performed a secondary analysis of data from participants of the National Health and Nutrition Examination Survey (NHANES) 2017-2018. The main exposure was HbA1c levels dichotomized into ≤ 7 and > 7. The primary outcome was Patient Health Questionnaire (PHQ-9) scores, dichotomized into no depression (scored 0-4 points) and depression regardless of severity (scored 5-27). Logistic regression was used to assess independent associations.

Results: Our sample included 429 adults with diabetes in the United States. About 41.5% had HbA1c > 7 and 26.8% presented some level of depression. The unadjusted analysis indicated that compared to adults with diabetes with HbA1c > 7, those with HbA1c ≤ 7 had 1.5 times greater odds of having some level of depression (OR = 1.5, 95% CI: 1.04-2.1, p-value = 0.033). However, in the analyses adjusted for sex, race/ethnicity, poverty, BMI, and sedentary lifestyle, the association between HbA1c levels and depression was no longer significant (OR = 1.2, 95% CI: 0.9-1.8, p-value = 0.256). Other factors increasing the odds of depression included lower income to poverty ratio ≤ 1.3 (OR 2.9, 95% CI: 1.0-8.5, p-value = 0.048) and sedentary lifestyle of 5-10 hours and >10 hours (OR = 2.7, 95% CI: 1.6-4.5, p-value = 0.001 and OR = 5.2, 95% CI: 1.7-15.4, p-value = 0.006, respectively).

Conclusion: Our study found no evidence for an association between HbA1c levels and depression. Due to limitations in power and the potential selection and measurement bias, further prospective studies in this field are needed. Implementation of depression screenings in people with diabetes may allow for timely treatment to those affected, improving the mental health of this population.

## Introduction

Diabetes mellitus is a group of complex metabolic disorders characterized by hyperglycemia [1]. In general, diabetes mellitus is associated with relative or absolute impairment of insulin secretion and varying degrees of peripheral resistance to insulin [2]. Type II diabetes mellitus is the most common type of diabetes, accounting for 90%-95% of all cases [3]. Type I diabetes mellitus, gestational diabetes, and diabetes due to other causes comprise the rest. Diabetes mellitus is a global epidemic affecting both developed and developing nations. The International Diabetes Federation (IDF) has estimated that the number of people with diabetes mellitus is 463 million worldwide. This number has doubled in the past two decades and is projected to reach 578 million by 2030 [4,5]. In the United States, the Centers for Disease Control and Prevention (CDC) estimates that diabetes mellitus affects 34.2 million people (10.5% of the population), of which 7.3% are undiagnosed [3]. By 2030, more than 55 million Americans are projected to have diabetes mellitus [6]. In 2017, the cost of diabetes mellitus was estimated to be $327 billion, of which $237 billion in direct medical costs and $90 billion in reduced productivity [7].

Diabetes mellitus is associated with devastating macrovascular and microvascular complications, such as cardiovascular disease, nephropathy, retinopathy, and neuropathy. These complications may lead to increased mortality, kidney failure, blindness, and overall decreased quality of life [8], thus optimal long-term glycemic control has been recommended [9,10]. Hemoglobin A1c (HbA1c) levels have been the primary method of monitoring long-term glycemia, reflecting the degree of hemoglobin glycation over the previous 8-12 weeks [11].

The Diabetes Attitudes, Wishes, and Needs (DAWN) study, a large multinational study that surveyed diabetic patients (type I and type II) and healthcare providers, revealed that 41% of patients with diabetes reported poor mental health [12]. Specifically, the prevalence of depression among people with diabetes is almost twice as high (17.6% vs. 9.8%) as compared to those without diabetes (9.8%) [13]. The World Health Organization estimates that more than 264 million people of all ages suffer from depression worldwide [14]. An economic burden, depression, is associated with more "years lost" to disability than any other condition [15]. In the United States, depression affects 8% of the population, accounting for more than $210 billion in costs [16].

Monitoring blood glucose levels could affect the mental health of patients with diabetes. At least two conflicting hypotheses exist for the potential directionality of the association between HbA1c and depression. One possibility is that higher HbA1c levels increase the risk for depression. The mechanism could be that higher HbA1c levels due to prolonged hyperglycemia indicate a high risk of diabetic complications, and thus those complications have been previously associated with poor mental health and depression [17]. Alternatively, there is a possibility that the risk of depression may be lower in those with higher levels of HBA1c. The mechanism for that would be that lowered HbA1c levels could be associated with more episodes of severe hypoglycemia [9]. Because there is an association between hypoglycemic events and poorer mental health and depression, our second hypothesis proposes that there is a connection between a lower HbA1c and a higher risk for depression [18].

Given the importance of HbA1c in monitoring and decision-making of diabetes mellitus, and given the connection established between diabetes mellitus and depression, our study aims to assess if there exists an association of HbA1c levels (as a proxy for glucose control) and depression occurrence among adults with diabetes mellitus in the United States. A better understanding of the potential added risk of depression to certain HbA1c levels is key for physicians managing diabetic patients. Better strategies for glucose monitoring and implementation of depression screenings may enable timely treatment for those affected and ultimately improve the mental health of this population.

## Materials and methods

Design and setting

This study was a cross-sectional study that analyzed data from participants of the National Health and Nutrition Examination Survey (NHANES) 2017-2018. Conducted by the CDC, the survey interviewed 9,254 individuals of all ages in their homes. The NHANES population is a representative sample of the noninstitutionalized civilian resident population of the United States. In the 2017-2018 survey, the NHANES oversampled certain subgroups of public health interest: Hispanic, non-Hispanic black, non-Hispanic Asian, non-Hispanic white, other persons at or below 185% of the Department of Health and Human Services poverty guidelines, and non-Hispanic white and other persons aged 80 years and older.

Sample

**Figure 1:**
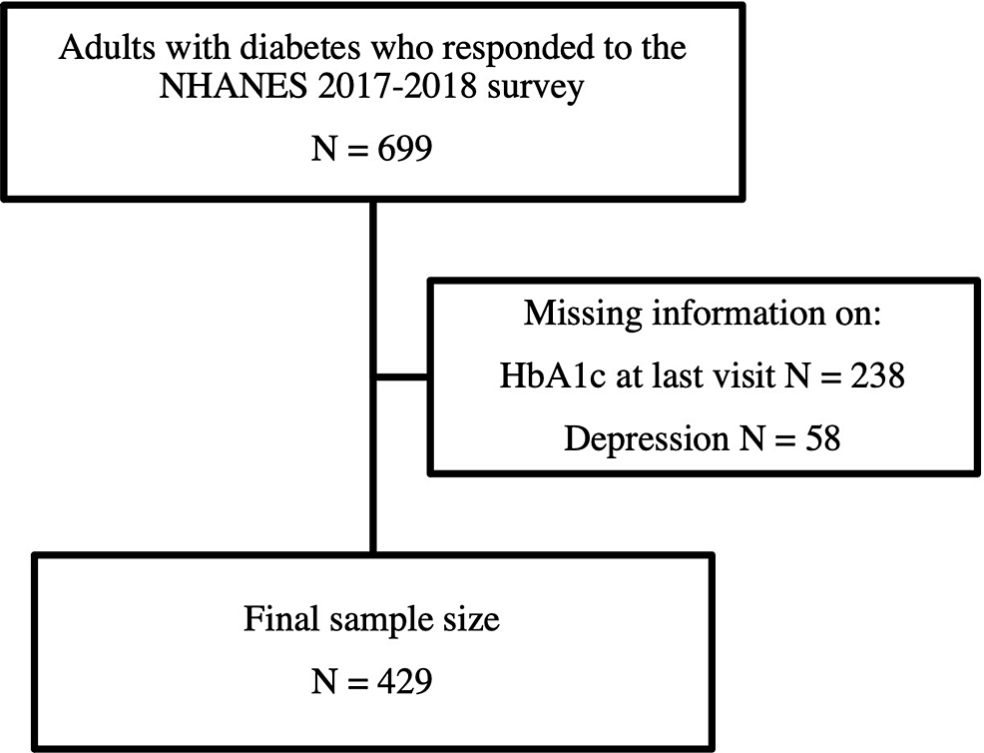
Study sample selection chart flow. NHANES: National Health and Nutrition Examination Survey; HbA1c: hemoglobin A1c.

Adults aged 18 years old or older with diabetes who completed both diabetes and depression questionnaires were included in the study. We excluded the pediatric population, patients with gestational diabetes, and patients with pre-diabetes or no diabetes mellitus (Figure 1).

Variables

The main independent variable of interest was the HbA1c level dichotomized into ≤7.0% and >7.0%. The cut-off was based on current American Diabetes Association guidelines for reasonable control of therapy [19]. The dependent variable was depression status according to the scores of the Patient Health Questionnaire (PHQ-9) screening scores. Results were then dichotomized into no depression (0-4 points) and mild/moderate/severe depression (5-27 points). Confounders assessed included sex, age, race/ethnicity, education, poverty, BMI, sedentary lifestyle, insulin use, family history of depression, poor diet, and medications.

Analytical plan

We used Stata version 16 (StataCorp LLC, College Station, TX) for all analyses, which included descriptive analyses, bivariate analyses to assess for potential confounders (variables with a difference of greater than 10% in magnitude or for which the corresponding p-value was <0.20), collinearity assessment, and finally multivariable logistic regression to assess for independent associations. The p-value for hypothesis testing was considered significant if p-value < 0.05.

## Results

Among the 9,254 individuals in the NHANES 2017-2018 survey, 699 were adults with diabetes. We were missing information on the depression score alone for 32 individuals, HbA1c level for 212 individuals, and both depression score and HbA1c for 26 individuals, so they were excluded from our population. This left a total of 429 adults with diabetes who answered information on HbA1c level and depression score in the survey.

Table 1 shows the baseline characteristics of the sample according to our dependent variable of interest. The sample was homogenous according to HbA1c levels with a few exceptions. The percentages of males, people of Hispanic origin, and with less education than a high school degree were higher in the group with an HbA1c > 7 than in the group with an HbA1c ≤ 7, while the percentage of those taking insulin was lower.

**Table 1:** Characteristics of the sample according to HbA1c levels. HbA1c: hemoglobin A1c; GED: General Educational Development; AA: associate of arts degree.

		HbA1c				
Patient characteristics		≤7%		>7%		P-value
		N^1 ^= 251	%^2^	N = 178	%	
Age	Less than 65 years	112	47.0	101	58.2	0.245
	65 years and older	139	53.0	77	41.8	
Sex	Male	136	49.8	103	66.9	0.005
	Female	115	50.2	75	33.1	
Race/ethnicity	White non-Hispanic	116	72.4	64	66.4	0.097
	Black non-Hispanic	48	8.0	40	9.4	
	Hispanic	29	5.9	42	13.7	
	Other/mixed	58	13.6	32	10.5	
Education	Less than high school	30	6.8	38	14.2	0.292
	High school/GED	60	26.0	36	22.2	
	Some college/AA	94	41.1	67	31.8	
	College graduate or higher	66	26.1	37	31.8	
Family income to poverty	≤1.30	46	13.2	40	14.8	0.757
	1.3-3.50	93	42.6	77	37.9	
	>3.50	84	44.2	50	47.3	
BMI	<25	33	7.4	15	7.8	0.689
	25.0-30	73	27.1	56	31.5	
	>30	143	65.5	103	60.7	
Sedentary lifestyle	<5 hours/day	95	32.2	64	32.5	0.664
	5-10 hours/day	135	49.6	87	53.1	
	>10 hours/day	31	18.2	27	14.4	
Taking insulin now	Yes	55	21.0	76	42.1	0.003
	No	196	79.0	102	57.9	

Table 2 shows the baseline characteristics of the sample according to our independent variable of interest. We found that women, those with higher levels of poverty, higher BMI, and those with a more sedentary lifestyle had the highest frequency of people in the higher depression score group.

**Table 2:** Characteristics of the sample according to depression status (PHQ-9 score categories). * A score of 0-4 was classified as no depression while a score of 5-27 was classified as depression (regardless of severity). PHQ-9: Patient Health Questionnaire; HbA1c: hemoglobin A1c; GED: General Educational Development; AA: associate of arts degree.

		PHQ-9 scores				
Patient characteristics		Depression		No depression*		P-value
		N = 314	%	N = 115	%	
HbA1c	≤7%	182	70.9	69	29.1	0.032
	>7%	132	78.2	46	21.8	
Age	Less than 65 years	149	74.1	64	25.9	0.930
	65 years and older	165	73.5	51	26.5	
Sex	Male	190	78.4	49	21.6	0.193
	Female	124	67.9	66	32.1	
Race/ethnicity	White non-Hispanic	119	71.9	61	28.1	0.459
	Black non-Hispanic	67	76.1	21	23.9	
	Hispanic	57	84.1	14	15.9	
	Other/mixed	71	75.6	19	24.4	
Education	Less than high school	46	68.0	22	32.0	0.429
	High school/GED	66	74.1	30	25.9	
	Some college/AA	114	70.3	47	29.7	
	College graduate or higher	87	80.1	16	19.9	
Family income to poverty	≤1.30	56	61.4	30	38.6	0.251
	1.3-3.50	118	70.2	52	29.8	
	>3.50	111	79.3	23	20.7	
Body mass index	<25	41	86.3	7	13.7	0.071
	25.0-30	101	80.0	28	20.0	
	>30	167	69.4	79	30.6	
Sedentary lifestyle	<5 hours/day	126	85.4	33	14.6	0.042
	5-10 hours/day	149	70.0	63	30.1	
	>10 hours/day	39	63.4	19	36.6	
Taking insulin now	Yes	87	71.1	44	28.9	0.491
	No	227	75.0	71	25.0	

In Table 3, we found that in the unadjusted analyses, there was a statistically significant association between an optimal level of HbA1c ≤ 7 and depression score (OR = 1.5, 95% CI = 1.0-2.1, p-value = 0.033). However, after adjusting for participants’ sex, race, poverty, BMI, and sedentary lifestyle, the association between HbA1c levels and depression was no longer significant (OR = 1.2, 95% CI = 0.9-1.8, p-value = 0.256).

**Table 3:** Associations between HbA1c levels and PHQ-9 scores. Note: Adjusted model included sex, race/ethnicity, poverty, body mass index, and sedentary lifestyle, all of which are shown in the table. PHQ-9: Patient Health Questionnaire; HbA1c: hemoglobin A1c.

Patient characteristics		Unadjusted		Adjusted	
		OR (95% CI)	P-value	OR (95% CI)	P-value
HbA1c	≤7%	1.47 (1.04, 2.09)	0.033	1.23 (0.85, 1.78)	0.256
	>7%	Ref		Ref	
Sex	Male	Ref		Ref	
	Female	1.72 (0.73, 4.03)	0.195	1.60 (0.71, 3.60)	0.239
Race/ethnicity	White non-Hispanic	Ref		Ref	
	Black non-Hispanic	0.81 (0.32, 2.05)	0.629	0.70 (0.30, 1.65)	0.388
	Hispanic	0.48 (0.22, 1.08)	0.073	0.41 (0.16, 1.03)	0.057
	Other/mixed	0.83 (0.30, 2.27)	0.692	1.11 (0.36, 3.45)	0.840
Family income to poverty	≤1.30	2.41 (0.81, 7.21)	0.11	2.93 (1.01, 8.50)	0.048
	1.3-3.50	1.63 (0.51, 5.15)	0.88	2.12 (0.69, 6.50)	0.175
	>3.50	Ref		Ref	
Body mass index	<25	Ref		Ref	
	25.0-30	1.58 (0.35, 7.12)	0.53	1.57 (0.33, 7.32)	0.545
	>30	2.78 (0.67, 11.51)	0.14	2.01 (0.52, 7.69)	0.286
Sedentary lifestyle	<5 hours/day	Ref		Ref	
	5-10 hours/day	2.51 (1.59, 3.94)	0.001	2.70 (1.58, 4.61)	0.001
	>10 hours/day	3.36 (1.17, 9.64)	0.027	5.16 (1.73, 15.40)	0.006

## Discussion

In our analysis, we found that 41.5% of all the individuals had an HbA1c level above 7, and 26.8% of our sample of individuals with diabetes had some level of depression. While there was a higher percentage of people who had depression in the group of HbA1c ≤ 7 (29.1% as compared to 21.8%), we did not find a significant association between levels of HbA1c and depression.

The prevalence of depression in our study (26.8%) was somewhat similar to that found in a meta-analysis that assessed the prevalence of depression in adults with diabetes [17]. In looking at 48 studies, the meta-analysis found that 31% of this population reported some level of depression. This meta-analysis used a variety of different tools (e.g., the Beck Depression Inventory or the Center for Epidemiologic Studies-Depression Scale) for depression screening, which may account for the small difference in the percentage of people with diabetes who experience depression.

A 2016 study assessed the prevalence of depression in American adults with type I diabetes using PHQ-8 (an adaptation of PHQ-9) [20]. They found a prevalence of 3.8-11.4% of depression, which was lower than the prevalence hereby reported. Like our study, this study did not find an association between HbA1c levels and depression. We suspect that the observed difference in the prevalence of depression might have been due to an exclusive type I diabetes sample, while our sample included both type I and type II diabetes. Given the prevalence of type II diabetes and the older age ranges in our sample, it is likely that the majority of our sample was made up of patients with type II diabetes. Thus, depression prevalence might be higher due to the older age range or potentially due to differing mechanisms influencing mental health in type I and II diabetes.

Another study of type I diabetes showed a relatively low prevalence (9%) of depression, which was defined as a score of 10 or higher on PHQ-9 [21]. Our study included a broader definition of depression (a score of 4 and higher on PHQ-9, which represents any level of depression). This, and the fact that this population included exclusively type I diabetics, may explain the difference between the prevalence in that study and ours. Unlike our study, this longitudinal study of 2,744 adults with type I diabetes did find an association between change in HbA1c from baseline to follow-up and depression status (p < 0.001 in all measured metrics). Moreover, since this is a longitudinal study (unlike our cross-sectional study) their results suggest directionality: an increase in the PHQ-8 (poorer mental status) leads to an increase in HbA1c levels (poorer glycemic control). The difference in associations found in this study and ours may be explained by differences in sample size (2,744 vs. 429) and sample composition (type I diabetes vs. type I and type II diabetes).

Similar to the results we reported in this study with respect to the association of HbA1c levels and depression (OR = 1.2, 95% CI = 0.9-1.8), a study of 514 Iranians with type II diabetes found no statistically significant association between depression (OR = 1.11, 95% CI = 0.87-1.57), as measured by the Beck Depression Inventory-II (BDI-II) tool, and HbA1c levels [22]. Because type II diabetes is the most prevalent type of diabetes accounting for >90% of cases, we believe that most of our sample consisted of type II diabetes, and thus, the sample of this study might resemble ours. Like our study, this study is limited by the small sample size and its cross-sectional nature. Important differences between the two studies include different HbA1c targets and the different depression screening tools used.

Our results somewhat contrast with the findings of another study that assessed depression among 2,718 adults with type II diabetes [18]. This study found that mean PHQ-9 scores were significantly higher among respondents who reported at least one hypoglycemic event compared to those who did not experience any (p < 0.001), indicating a possible greater depression burden. Hypoglycemic events are also more common among tighter control of diabetes as measured by HbA1c [23]. A potential explanation for the difference between the results of this study and the results from ours is that our results' lack of significance might be due to the small sample size.

Our study has limited power (n = 429). Further, because of our small sample size, all severity levels of depression were grouped together (mild, moderate, and severe), which precluded differentiation among groups of those who have mild, moderate, or severe depression based on their HbA1c levels. Given that the data collected were from a cross-sectional study, we are also unable to assess the temporality of our dependent and independent variables. Thus, we cannot determine if HbA1c levels would lead to depression or if depression status would lead to different levels of HbA1C. Lastly, our study may have been subject to bias. First, a selection bias among the population we sampled, as 34% of participants were missing information on HbA1c. We do not know into what depression categories the individuals would potentially be placed into and therefore, it affects the internal validity of our study. Second, the survey used for depression, PHQ-9, is a self-administered questionnaire and could be subject to under-reporting of the outcome, which could potentially have affected our power. However, PHQ-9 is considered a valid and more accurate depression screen than others with a sensitivity of 88% and specificity of 88% [24].

## Conclusions

In conclusion, the prevalence of depression among adults in the United States with diabetes was high (26.8%), but we found no evidence for an association between HbA1c levels and depression status. Yet, these results should be taken in light of the limitations. Given the high prevalence of depression among diabetics, the implementation of depression screenings to diabetics at high risk for depression may provide the opportunity for timely treatment to those affected and ultimately improve the mental health of this population.

## References

[1] Roden M (2012). Diabetes mellitus: definition, classification and diagnosis. (Article in German). Wien Klin Wochenschr.

[2] Silvio E Inzucchi, Beatrice Lupsa (2021). Clinical presentation, diagnosis, and initial evaluation of diabetes mellitus in adults. https://www.uptodate.com/contents/clinical-presentation-diagnosis-and-initial-evaluation-of-diabetes-mellitus-in-adults.

[3] (2020). CDC. National diabetes statistics report. National Diabetes Statistics Report.

[4] Zimmet P, Alberti KG, Magliano DJ, Bennett PH (2016). Diabetes mellitus statistics on prevalence and mortality: facts and fallacies. Nat Rev Endocrinol.

[5] International Diabetes Federation (2021). International Diabetes Federation. IDF Diabetes Atlas. https://diabetesatlas.org/.

[6] Rowley WR, Bezold C, Arikan Y, Byrne E, Krohe S (2017). Diabetes 2030: insights from yesterday, today, and future trends. Popul Health Manag.

[7] American Diabetes Association (2018). Economic costs of diabetes in the U.S. in 2017. Diabetes Care.

[8] Cole JB, Florez JC (2020). Genetics of diabetes mellitus and diabetes complications. Nat Rev Nephrol.

[9] Nathan DM, Genuth S, Lachin J (1993). The effect of intensive treatment of diabetes on the development and progression of long-term complications in insulin-dependent diabetes mellitus. N Engl J Med.

[10] UK Prospective Diabetes Study (UKPDS) Group (1998). Effect of intensive blood-glucose control with metformin on complications in overweight patients with type 2 diabetes (UKPDS 34). Lancet.

[11] Bădescu SV, Tătaru C, Kobylinska L, Georgescu EL, Zahiu DM, Zăgrean AM, Zăgrean L (2016). The association between diabetes mellitus and depression. J Med Life.

[12] Peyrot M, Rubin RR, Lauritzen T, Snoek FJ, Matthews DR, Skovlund SE (2005). Psychosocial problems and barriers to improved diabetes management: results of the Cross-National Diabetes Attitudes, Wishes and Needs (DAWN) study. Diabet Med.

[13] Ali S, Stone MA, Peters JL, Davies MJ, Khunti K (2006). The prevalence of co-morbid depression in adults with type 2 diabetes: a systematic review and meta-analysis. Diabet Med.

[14] (2021). World Health Organization. Depression. https://www.who.int/news-room/fact-sheets/detail/depression.

[15] Smith K (2014). Mental health: a world of depression. Nature.

[16] Maurer DM, Raymond TJ, Davis BN (2018). Depression: screening and diagnosis. Am Fam Physician.

[17] Anderson RJ, Freedland KE, Clouse RE, Lustman PJ (2001). The prevalence of comorbid depression in adults with diabetes: a meta-analysis. Diabetes Care.

[18] Green AJ, Fox KM, Grandy S (2012). Self-reported hypoglycemia and impact on quality of life and depression among adults with type 2 diabetes mellitus. Diabetes Res Clin Pract.

[19] American Diabetes Association (2013). Standards of medical care in diabetes—2013. Diabetes Care.

[20] Fisher L, Hessler DM, Polonsky WH, Masharani U, Peters AL, Blumer I, Strycker LA (2016). Prevalence of depression in type 1 diabetes and the problem of over-diagnosis. Diabet Med.

[21] Trief PM, Foster NC, Chaytor N (2019). Longitudinal changes in depression symptoms and glycemia in adults with type 1 diabetes. Diabetes Care.

[22] Mansori K, Shiravand N, Shadmani FK (2019). Association between depression with glycemic control and its complications in type 2 diabetes. Diabetes Metab Syndr.

[23] American Diabetes Association (2018). Glycemic targets: standards of medical care in diabetes—2018. Diabetes Care.

[24] Kroenke K, Spitzer RL, Williams JB (2001). The PHQ-9: validity of a brief depression severity measure. J Gen Intern Med.

